# Post Weld Heat Treatment Optimization of Dissimilar Friction Stir Welded AA2024-T3 and AA7075-T651 Using Machine Learning and Metaheuristics

**DOI:** 10.3390/ma16052081

**Published:** 2023-03-03

**Authors:** Pinmanee Insua, Wasawat Nakkiew, Warisa Wisittipanich

**Affiliations:** 1 Graduate Program in Industrial Engineering, Faculty of Engineering, Chiang Mai University, Chiang Mai 50200, Thailand; 2 Department of Industrial Engineering, Faculty of Engineering, Chiang Mai University, Chiang Mai 50200, Thailand; 3 Advanced Manufacturing and Management Technology Research Center (AM2Tech), Department of Industrial Engineering, Faculty of Engineering, Chiang Mai University, Chiang Mai 50200, Thailand

**Keywords:** dissimilar friction stir welding, post weld heat treatment, machine learning, metaheuristics, process optimization, ultimate tensile strength, elongation percentage

## Abstract

Post weld heat treatment, or PWHT, is often used to improve the mechanical properties of materials that have been welded. Several publications have investigated the effects of the PWHT process using experimental designs. However, the modeling and optimization using the integration of machine learning (ML) and metaheuristics have yet to be reported, which are fundamental steps toward intelligent manufacturing applications. This research proposes a novel approach using ML techniques and metaheuristics to optimize PWHT process parameters. The goal is to determine the optimal PWHT parameters for both single and multiple objective perspectives. In this research, support vector regression (SVR), K-nearest neighbors (KNN), decision tree (DT), and random forest (RF) were ML techniques employed to obtain a relationship model between PWHT parameters and mechanical properties: ultimate tensile strength (UTS) and elongation percentage (EL). The results show that the SVR demonstrated superior performance among ML techniques for both UTS and EL models. Then, SVR is used with metaheuristics such as differential evolution (DE), particle swarm optimization (PSO), and genetic algorithms (GA). SVR-PSO shows the fastest convergence among other combinations. The final solutions of single-objective and Pareto solutions were also suggested in this research.

## 1. Introduction

Friction stir welding (FSW) was invented in 1991 at The Welding Institute (TWI) of the UK as a solid-state joining process [1]. FSW is extensively applied to high-strength aluminum alloys, especially the alloyed AA2XXX and AA7XXX series, which are widely used in important manufacturing industries such as automobiles, aircraft, aerospace, and military industries due to their high strength, low density, high fracture toughness, good electrical conductivity, and corrosion resistance [2]. FSW results in intense plastic deformation around the rotating tool and friction between the tool and workpieces. Both of these factors increase the temperature within and around the stirred zone and generate a softened region within the weld zone because of the dissolution of strengthening precipitates [1,3]. Therefore, as a consequence, precipitation-hardening aluminum alloy joints generally have a lower weld strength than base metal [4,5]. Many techniques are being considered to improve the properties of weld joints, such as welding process parameter optimization [6,7,8] and post weld heat treatment (PWHT) [9,10,11,12,13,14,15]. Güven et al. [15] compared the mechanical properties of friction stir welded AA6061 plates before and after applying PWHT. The result showed that the joint performance values of PWHT specimens in terms of proof stress, tensile strength, and elongation were slightly better at 84, 88, and 44 pct, respectively. Hakan et al. [9] also observed that the PWHT process increased the tensile strength efficiency and yield strength efficiency of the as-welded joint to 87.4% and 98%, respectively. Chaitanya et al. [10] examined the influence of PWHT on microstructure and mechanical properties. PWHT increased the size of aluminum grains in all zones of friction stir weld joints, and naturally aged joints offered the best mechanical properties. Sivaraj et al. [11] also reported that after solution treatment and artificial aging, the yield strength and tensile strength of friction stir-welded AA7075 aluminum alloy joints were increased to 346 MPa and 445 MPa, respectively, increasing joint efficiency by 9% over as-welded joints. According to the literature review, the PWHT process parameters had a direct impact on the properties of welded joints, such as tensile strength, hardness, residual stress, and toughness. For example, Vinay Kumar et al. [14] demonstrated that PWHT significantly improved the ductility and the hardness of the welded joint at 200 °C for 10 h compared to the as-welded joint and the PWHT joint at 190 °C for 10 h. In addition, we found that several researchers [16,17,18,19] studied the influence of the PWHT process on the same metal, but they used different parameters. Hence, many researchers intend to optimize the parameters of the PWHT process. Chin Chun et al. [20] used the Taguchi experimental design method to analyze optimal heat treatment parameters for the 7050 aluminum alloy. Mehmet et al. [21] also investigated the effect of PWHT parameters with the Taguchi method and used the genetic algorithm for optimization. It clearly shows that the function of PWHT is still implemented by using traditional methods such as trial-and-error, design of experiments (DOE), response surface methodology (RSM), or the Taguchi method. In contrast, the other manufacturing process uses more intelligent approaches such as AI and machine learning (ML) techniques to obtain the advanced process.

The ML technique is the science of teaching machines to learn on their own to solve real-time problems based on input data. Recently, ML has been applied in many research fields and has continuously grown with more authors to increase computer systems’ intelligence [22]. In manufacturing, the applications of ML refer to pattern recognition in existing sets of data for forecasting the future behavior of the system [23] and provide the promising potential to improve quality control optimization in the manufacturing process [24]. For example, Kaneko and Funatsu [25] made an application of the online SVR model for soft sensor development. He and Wang [26] developed a statistical pattern analysis and process monitoring method based on the K-nearest neighbors (KNN) method. Tauqir et al. [27] studied the applications of ML to FSW process optimization. The results show that artificial neural networks (ANN) outperformed RSM in terms of robustness and accuracy. In addition, they found that the DOE provided a lack of a systematic approach to process parameters. In the phase of cloud manufacturing [28] and smart manufacturing, known as industry 4.0, currently, ML has the ability to become the main driver in uncovering fine-grained, intricate production patterns in the smart manufacturing paradigm [29]. On the other hand, we found no application of ML models to implement for the PWHT experiment in the literature review, because the PWHT process is difficult to test on a large scale, time-consuming, and expensive. These problems are consistent with the contribution of ML techniques to solving problems and increasing intelligence.

SVR, KNN, decision tree (DT), and random forest (RF) are examples of supervised machine learning algorithms used in manufacturing [30]. The goal of these techniques is to produce a predicted model based on the training data. In this study, four ML techniques are employed to create a model of the PWHT process to determine the relationship between parameters and two mechanical properties, such as ultimate tensile strength and elongation percentage. Then, the process parameter optimization is carried out using metaheuristic algorithms. Differential evolution (DE), particle swarm optimization (PSO), and genetic algorithms (GA) are population-based metaheuristic approaches that are popularly used in the field of manufacturing process optimization [21,31,32,33,34]. Ezra and Weihang [35] reported that GA and DE are more common for solving both process and system optimization problems among the 116 papers in their survey of the food manufacturing industry. Nizar et al. [36] demonstrated that PSO, real-coded GA, and DE obtained the same optimum for friction stir welding parameter optimization, but PSO had a faster convergence than the other algorithms. Ricardo et al. [37] discovered that PSO provided a better solution than GAs for a given micro-milling operation. For solving both single- and multi-objective problems in this research, DE, PSO and GA were executed. The specific contributions of this research are as follows:

- A novel approach using ML techniques and a metaheuristic algorithm is proposed for solving single- and multi-objective problems of PWHT process parameter optimization.
- The relationship between parameters and mechanical properties in the PWHT process of dissimilar friction stir welded 2024-T3 and 7075-T651 aluminum alloys are investigated using ML techniques to model single and multi-objective.
- In this study, the use of ML techniques combined with metaheuristics encourages the intelligent manufacturing process, particularly the PWHT process, to be more adaptive, real-time, faster, and cost-effective.

## 2. Materials and Methods

### 2.1. Workpiece Material

In this study, AA7075-T651 and AA2024-T3 with dimensions 200 mm × 100 mm × 3 mm were used for the experiment. The chemical compositions of two materials were measured by the energy-dispersive X-ray fluorescence (EDXRF) method using the JEOL model JSX3400R, as shown in Table 1.

### 2.2. Friction Stir Welding

**Figure 1:**
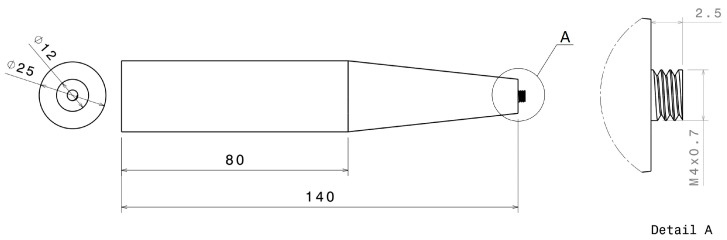
A schematic dimension and geometry of the cylindrical thread tool (M4 × 0.7 thread designation: pitch = 0.7 mm, major diameter = 4 mm).

**Figure 2:**
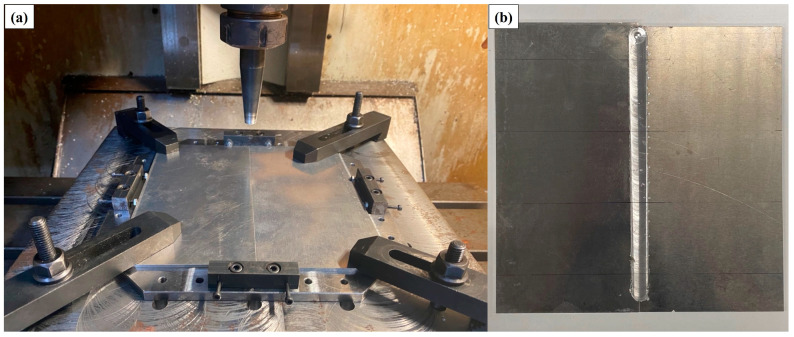
(**a**) Setup of the experiment; (**b**) a welded aluminum alloy plate.

The FSW was used to fabricate dissimilar aluminum alloy plate joints between AA7075-T651 and AA2024-T3. The tool was made of high-carbon steel SKD61 and heat-treated to 55 HRC. The dimensions of the welding tool are in millimeters, as shown in Figure 1. The welding parameters were set at a rotational speed of 1200 rpm, a welding speed of 100 mm/min [38], and a plunge depth of 0.2 mm. The support system and welding setup are shown in Figure 2.

### 2.3. Post Weld Heat Treatment (PWHT)

After the welding process, a joined plate was cut into three parts in the same transverse direction as the welding specimens, and their dimensions were 200 mm × 100 mm × 3 mm. A full factorial design of 2^4^ with 4 center points and 2 replications, resulting in 36 experiments, was implemented. Each part was tested in different conditions of the PWHT process parameters, as shown in Table 2. The workpieces were heated to the solid solution temperature and soaked in a hardening furnace (Manufacturer: Nabertherm, Germany) in the first stage. Model: N41/H). Subsequently, the heated workpieces were transferred within 10 s and quenched in water to obtain a supersaturated solid solution state at different temperatures. After the cooling operation, the quenched parts were artificially aged to reach full strength.

### 2.4. Tensile Test

**Figure 3:**
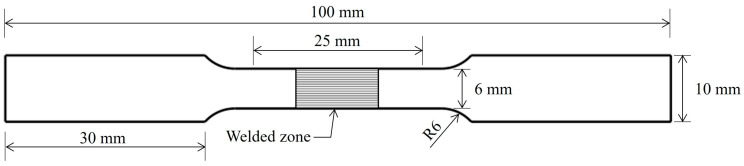
Dimensions of tensile test specimen.

**Figure 4:**
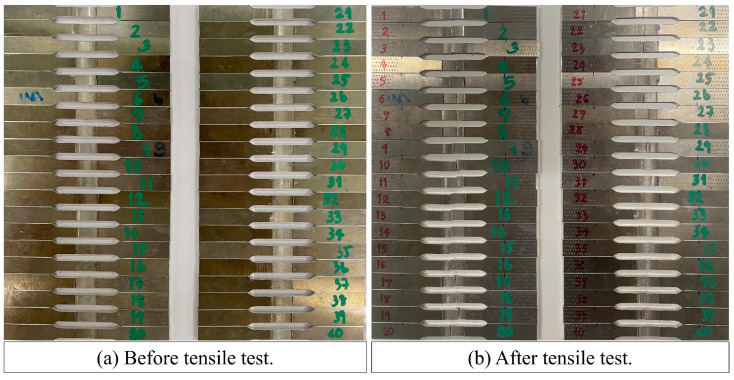
Photograph of tensile test specimens.

The workpieces were machined to the dimensions of a tensile test specimen following the ASTM B 557 M standard (25 mm gauge length), as shown in Figure 3. The tensile test was conducted using a universal testing machine from Cometech, model QC-506. The test rate was 5 mm/min. The ultimate tensile strength (UTS) was evaluated, and the elongation percentage (%EL) was calculated. Figure 4a,b show photographs of tensile test specimens before and after the test.

### 2.5. Modeling by Machine Learning Techniques

**Figure 5:**
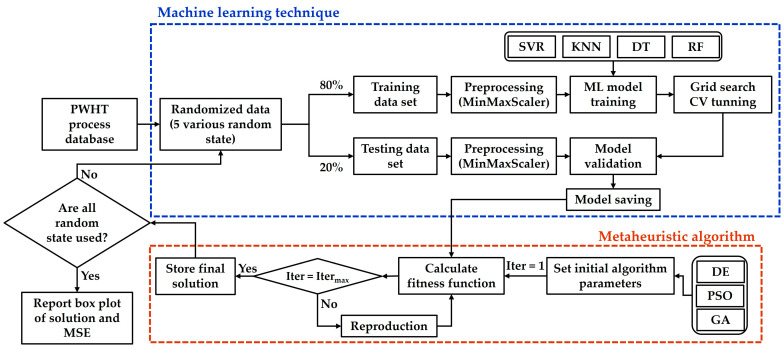
Conceptual workflow of the machine learning techniques.

In this section, the Python programming language was executed on Spyder (Anaconda 3) software to subject the supervised machine learning algorithms to testing. For preprocessing, the MinMaxScaler was used to normalize the input features into a range [0, 1]. In this study, 28 out of 36 datasets (approximately 80%) were used as training datasets in machine learning models. The results suggested the most suitable hyperparameters using Grid Search Cross Validation (CV = 3). Then, 8 out of 36 datasets (approximately 20% of the dataset) were used to evaluate the model. Five variations in the random state were applied for all runs and repeated three times. The model’s prediction performance was measured by the statistical MSE. The conceptual workflow in Figure 5 shows the steps of the ML technique and the connection point between ML and metaheuristic algorithms. First, the database collected from the PWHT experiment was split into training and testing datasets and normalized. Then, using the ML technique learned from the training dataset, a model was constructed using grid search CV to tune the hyperparameters. The model was validated by testing a dataset and saved. In the optimization process, the model from ML was used as a fitness function. The process started with setting the initial algorithm parameters to Iter (iteration) = 1 before calculating the fitness value. If Iter < Iter_max_, the fitness value was updated and reproduced. If Iter = Iter_max_, then the next step proceeded. The final solution of a random state was stored. It was checked whether all random states were used or not. If not, the first step was returned to, and the process was restarted.

### 2.6. Process Parameters Optimization

#### 2.6.1. Single-Objective Optimization

In this research, differential evolution (DE), particle swarm optimization (PSO), and genetic algorithms (GA) were applied to parameter optimization. The fitness function was conducted using the ML technique as Equations (1) and (2). For all algorithms, population and maximum iteration were set to 50 and 300, respectively. Due to four input factors, the search space was limited to four dimensions. The upper and lower bounds of solid solution time (*A*), solid solution temperature (*B*), aging time (*C*), and aging temperature (*D*) are presented as follows:


(1) $$\mathrm{Maximize}:\;f_{1}(A,B,C,D)\;=\;\mathrm{predicted}\mathrm{function}\mathrm{of}\mathrm{UTS}\mathrm{obtained}\mathrm{from}\mathrm{machine}\mathrm{learning}\mathrm{technique}.$$

(2) $$\mathrm{Maximize}:\;f_{2}(A,B,C,D)\;=\;\mathrm{predicted}\mathrm{function}\mathrm{of}\mathrm{EL}\mathrm{obtained}\mathrm{from}\mathrm{machine}\mathrm{learning}\mathrm{technique}.$$

Subject to:

A 0.5 h ≤ solid solution time (*A*) ≤ 4 h;

A 460 °C ≤ solid solution temperature (*B*) ≤ 480 °C;

A 6 h ≤ aging time (*C*) ≤ 24 h;

A 120 °C ≤ aging temperature (*D*) ≤ 190 °C.

#### 2.6.2. Multi-Objective Optimization

In several multi-objective optimizations, it is difficult to simultaneously provide a satisfying solution for different objectives. Therefore, a possible answer for multi-objective optimization is a set of solutions called Pareto-optimal solutions or non-dominated solutions. Hajela and Lin [39] introduced a popular approach that is based on simple aggregation. They use the weighted aggregation method for fitness assignments. This approach is easy to use for solving multi-objective problems. Weight factors were varied to obtain all solutions of a combination and show the different relations of two objective functions.

1. Weighted aggregation method

The most common method to convert a multi-objective problem into a single objective is using weight factors. Based on this research, the two objective functions were summed up with varying weights, giving Equation (3).

(3) $$\mathrm{maximize}:\;F(X)\;=\;w_{1}f_{1}(X)\;+\;w_{2}f_{2}(X)$$

where

$f_{1}$ and $f_{2}$ represent two objective functions;

*X* is the decision variables;

$w_{1}$ and $w_{2}$ are the weight factors.

Define $w_{1}\;=\;k/N$. The combined objective function in Equation (3) can be rewritten as

(4) $$F(X)\;=\;\frac{k}{N}f_{1}(X)\;+\;\frac{N-k}{N}f_{2}(X)$$

subject to

$w_{1}$ + $w_{2}$ = 1, $w_{1}\;\geq \;0$, and $w_{2}\;\geq \;0$

where *N* is the population size, and *k* starts from 1 to *N* (use *N* = 20).

The framework of the weight sum method is described as Algorithms 1 follows.

**Algorithms 1**: Weighted Aggregation Method.1. initialize population size *N* and other parameters2. set *k* = 13. Loop      While *k* < *N*            (1) Set *w* = *k/N*            (2) Execute a single objective optimization algorithm to find the final value using Equation (4)            $wf_{1}(X)+(1-X)f_{2}(X)$            (3) Store the information on the solution            (4) *k* = *k* + 1      End4. Plot the Pareto front

2. Hypervolume indicator

**Figure 6:**
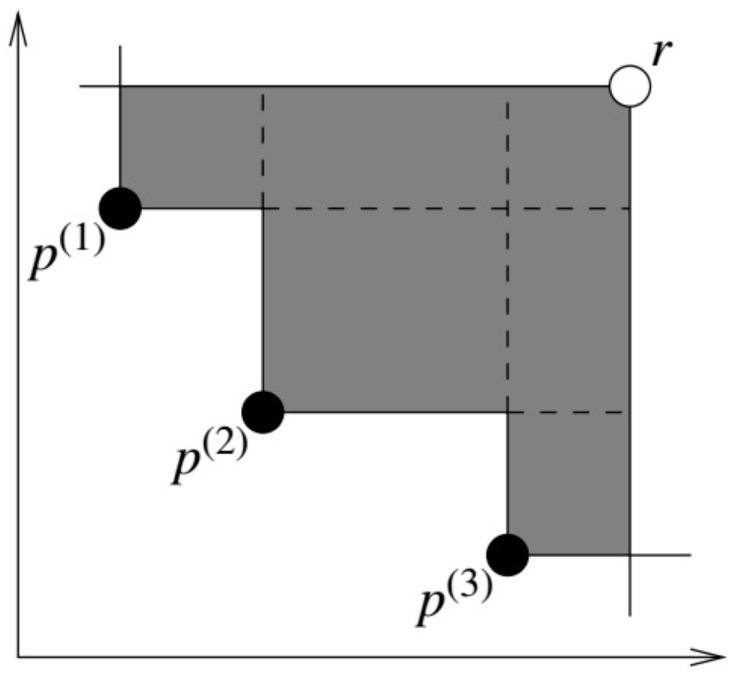
The hypervolume indicator in the two objectives [41].

The hypervolume indicator [40] is a measure of the quality of a set $p=\{p^{\left(1\right)},p^{\left(2\right)},\ldots ,p^{\left(n\right)}\}$ of *n* nondominated objective vectors produced by the multi-objective optimizer. This indicator consists of the measure of the region dominated by *P* and bounded above by a reference point $r\in R^{d}$, such that $r\geq (\max_{p}p_{1},\ldots ,\max_{p}p_{d})$, where $p=(p_{1},\ldots ,p_{d})\in P\subset R^{d}$, and the relation ≥ applies component wise [41], as shown in Figure 6. In this work, the performance of each metaheuristic algorithm was measured by computing the hypervolume metric and comparing it with the region’s size.

## 3. Results and Discussion

As mentioned, the full factorial design (2^4^ with four center points and two replications) was used in the experiment. The two responses, UTS and EL, are reported in Table 3.

**Figure 7:**
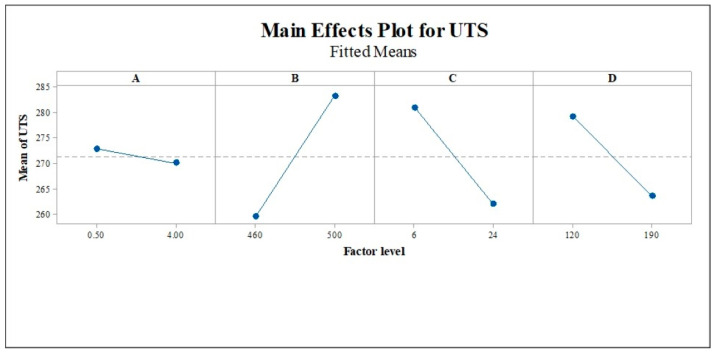
Main effect plot for the UTS response. (A) = solution time, (B) = solution temperature, (C) = aging time, and (D) = aging temperature.

The analysis of variance (ANOVA) technique used statistical data analysis to provide a significant process parameter for the experimental results of the ultimate tensile strength (UTS) and elongation percentage (EL). In this study, an ANOVA was executed on Minitab 18 with a significant level of 5%, i.e., a confidence level of 95%. When the *p*-value is less than 0.05, the significance of the process parameters to the response value is acceptable. The ANOVA result of UTS in Table 4 shows that three parameters, solid solution temperature (B), aging time (C), and aging temperature (D), had significant effects, but solid solution time (A) was insignificant due to a *p*-value of >0.05 [42]. The two-way, three-way, and four-way interactions are also demonstrated to be substantial. As shown in Figure 7, the main effect plot reveals that higher UTS can be obtained with high solution temperatures. Increasing the solution time, aging time, and temperature reduces UTS, as observed by Polmear and Couper [43].

**Figure 8:**
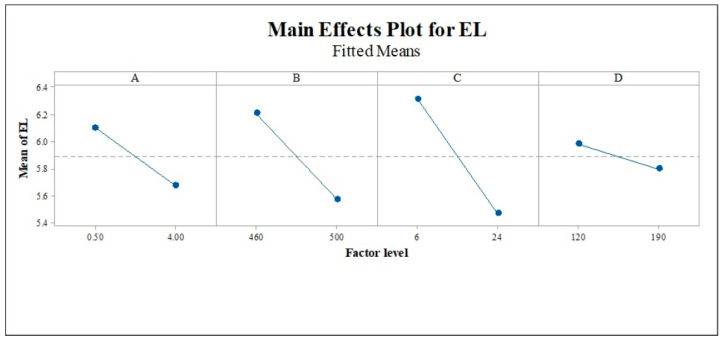
Main effect plot for the EL response. (A) = solution time, (B) = solution temperature, (C) = aging time, and (D) = aging temperature.

An ANOVA result for the EL response is shown in Table 5. Three parameters, solution time (A), solution temperature (B), and aging time (C), have significant effects with *p*-values smaller than 0.05. According to P. Sivaraj et al. [44], decreasing the aging time reduced the elongation percentage. The reduction in ductility is attributed to the abnormal grain growth and coarsening of the hardening precipitate. Some two-way and three-way interactions also demonstrate significance, but none of the four-way interactions. The main effect plot, as shown in Figure 8, determines the influence of each parameter on the elongation percentage. From Table 4 and Table 5, the *p*-values of the UTS and EL models are smaller than 0.05. It means the parameters are significant to construct models for UTS and EL prediction.

**Figure 9:**
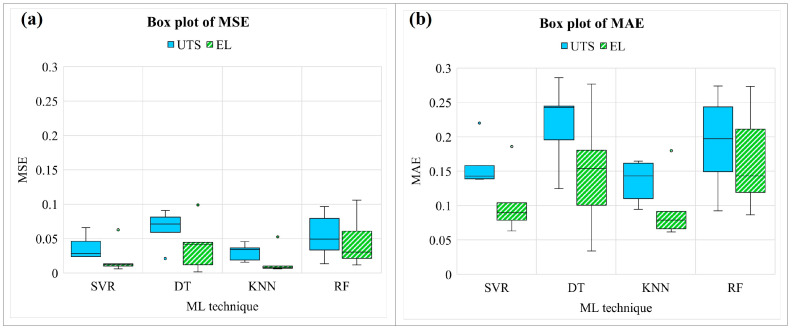
Box plot of training and testing MSE for UTS and EL model.

In the model development section, the experimental PWHT process dataset is smaller than the general problem. Therefore, the consistency of the ML performance in a different random state and the effect of randomization on the dataset were considered by using five variations in random states. Grid search cross-validation was applied to tune hyperparameters for the ML technique, and the final values are shown in Table 6, Table 7, Table 8 and Table 9. The ML technique used different hyperparameters in different random states. Since ML strongly depends on the input dataset, tuning is always applied before prediction. The box plot of the MSE and MAE for different ML techniques is shown in Figure 9, and the average values are shown in Table 10. For the UTS model, the average MSE of SVR, DT, KNN, and RF are 0.03748, 0.06455, 0.03, and 0.05591, respectively. For the EL model, the average MSE of SVR, DT, KNN, and RF are about 0.02072, 0.03971, 0.01659, and 0.04228, respectively. It demonstrates that SVR and KNN build the UTS model and EL model with the lowest average MSE and MAE, whereas DT and RF produce more MSE with a larger range of box plots.

**Figure 10:**
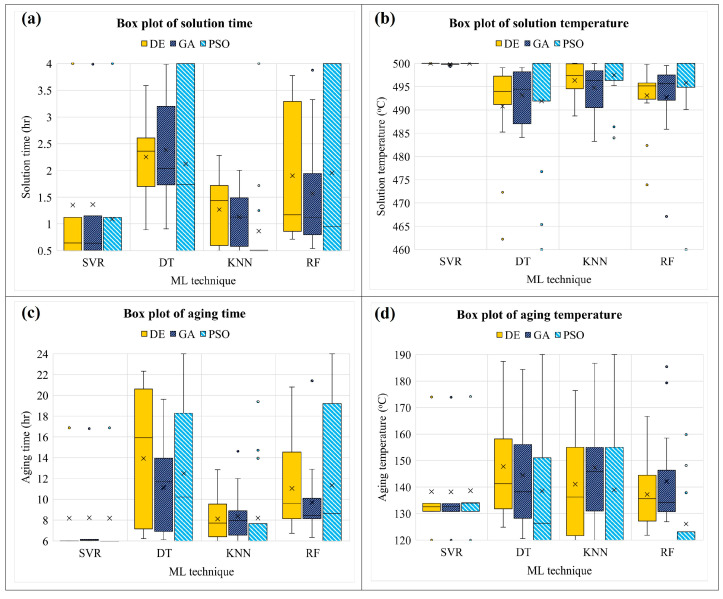
Box plots of the final solutions of the UTS model.

**Figure 11:**
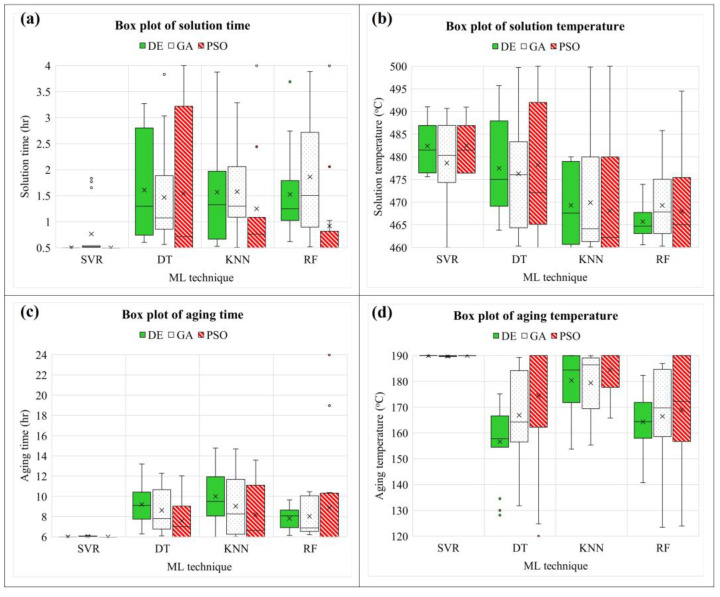
Box plots of the final solutions of the EL model.

**Figure 12:**
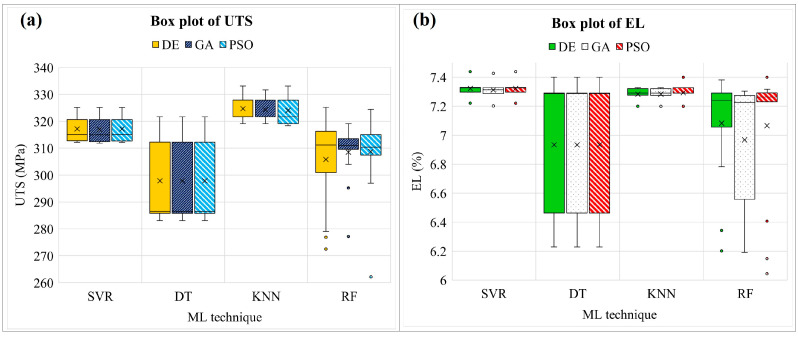
Box plots of the final value of UTS and EL.

In this study, UTS and EL models were used as objective functions, with solid solution time (A), solid solution temperature (B), aging time (C), and aging temperature (D) as decision variables. The ML-based model is experimentally combined with a metaheuristic algorithm to optimize the parameters of the PWHT process and propose the appropriate combination. After a combination is run with various random states, the value of the final solution and responses are shown in a type of box plot. For the UTS model, it is observed that SVR is outstanding in giving a robust model due to providing the smallest range of box plots, as shown in Figure 10. Similar to the EL model, SVR also offers a model that does not become stuck in the local optimum during optimization [45,46] while changing the training dataset (random state) and usually gives almost the same final solution when optimization is complete, as shown in Figure 11. It also found in Figure 12a,b, SVR provide the smallest range of box plots of the final value. In contrast, DT, KNN, and RF produce an extensive range of solutions, especially DT-PSO and RF-PSO. Thus, SVR is the most suitable model based on the given data in this research, and similar results were observed by Wuest et al. [47].

**Figure 13:**
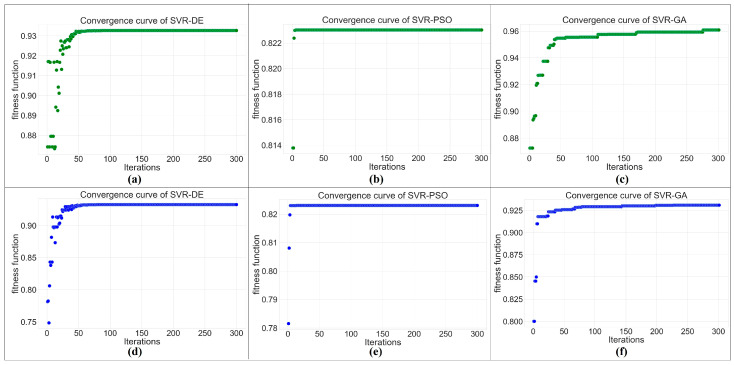
The convergence curve of various combinations: (**a**) SVR-DE, (**b**) SVR-PSO, (**c**) SVR-GA for the UTS model; (**d**) SVR-DE, (**e**) SVR-PSO, (**f**) SVR-GA for the EL model.

Table 11 shows the value of the final solution (decision variables) and objective functions obtained by combinations of SVR and metaheuristics. When SVR works with DE and PSO algorithms, the final solutions of both UTS and EL models are the same. Additionally, GA is slightly different. The UTS can be maximized by solid solution annealing at 500 degrees Celsius for 30 min and artificial aging at 133.87 degrees Celsius for 6 h. Moreover, solid solution time, solid solution temperature, aging time, and aging temperature are suggested to be set at 30 min, 476.44 °C, 6 h, and 190 °C, respectively, for maximizing EL. To consider the convergent performance of the model, Figure 13a–f shows a graphical plot between the best fitness values and iterations. According to Figure 13b,e, PSO converges faster than other algorithms following DE and GA, respectively.

**Figure 14:**
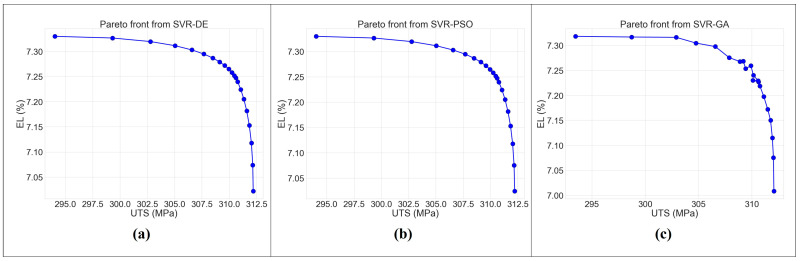
The Pareto solution for multi-objective functions from (**a**) SVR-DE, (**b**) SVR-PSO, and (**c**) SVR-GA.

SVR was chosen for multi-objective analysis combined with metaheuristic algorithms based on its best performance on given PWHT process data in this study. Two objective functions obtained from SVR were converted into a single one by using weighted aggregation. However, the important issue in the method is the weight assignment because the solution strongly depends on the chosen weighting coefficients. Therefore, the Pareto solution was proposed to fix the problem. The result of a multi-objective optimization yields sets of trade-off solutions between UTS and EL. As shown in Figure 14, the Pareto front shows that the elongation percentage decreases with the increase in the ultimate tensile strength, as reported by [48]. Furthermore, the main advantage of the proposed multi-objective is that it provides sets of solutions with a variety of weighting coefficients from which to select the appropriate one to apply to the specific user-defined process as alternative solutions.

In order to measure the distinction between the Pareto solutions, the hypervolume indicator was applied to quantify the quality of a solution set [49] using a single reference point r = (330,7.5). This measure aims to minimize the volume between solution sets and the reference point. It is observed from Figure 14 that the regions of hypervolume are concave due to optimizing the hypervolume in maximization problems [40,50]. The hypervolumes of SVR-DE, SVR-PSO, and SVR-GA are 12.004, 12.043, and 12.607, respectively. Table 12 shows that the SVR-DE has the smallest volume when compared to the others. It means the solution sets of SVR-DE can better reach the maximum value of two objective functions. However, the SVR-PSO also has a small volume, almost the same as the SVR-DE, whereas the SVR-PSO converges to the final solutions faster.

## 4. Conclusions

The PWHT process was conducted for dissimilarly FSWed 2024-T3 and 7075-T651 aluminum alloys. According to the experimental results and analysis carried out, it was concluded that the parameters in the experimental design significantly affected mechanical properties such as UTS and EL. The following conclusions can be drawn based on the results and discussion:

The SVR model demonstrated superior performance among ML techniques for both UTS and EL models based on the given data in this study.

SVR-DE and SVR-PSO reached the same final solution for the UTS and EL models, but SVR-PSO shows the fastest convergence among other combinations. 

The final solutions showed that the UTS could be increased by treating the solid solution at 500 °C for 30 min and aging it artificially at 133.87 °C for 6 h. For maximized EL, solid solution time, solid solution temperature, aging time, and aging temperature were set at 30 min, 476.44 °C, 6 h, and 190 °C, respectively.

The Pareto front was proposed as an alternative solution with a variety of weighting coefficients.

SVR-DE gave the smallest volume of 12.004, followed by SVR-PSO and SVR-GA with 12.043 and 12.607, respectively. It was concluded that the solution sets from SVR-DE can reach the maximum value of two objective functions more than SVR-PSO and SVR-GA.

In conclusion, this research presented contributions to developing the PWHT process in terms of model prediction and parameter optimization by using the ML technique, which is one of the main characteristics of smart manufacturing design and is expected to progress quality control. In machine learning techniques, the data used for training are not limited to the designed level like in experimental techniques. This makes the process of collecting data more flexible. When compared to the traditional design of an experiment, this would be the primary advantage. In addition, the metaheuristic approach in this study provides trade-off solutions for multi-objective problems with a variety of suitable options for different decision-makers. Further work can be applied with the finite element method (FEM) to study real-time material properties and add the significant factors of the PWHT process to obtain the results with a high-dimensional and more intelligent program.

## Figures and Tables

**Table 1:** Chemical composition of AA7075-T651 and AA2024-T3 aluminum alloys (% *w*/*w*).

Elements	Al	Zn	Mg	Cu	Mn	Cr	Si	Fe	S	Other
AA7075-T651	82.50	9.20	3.50	2.23	0.08	0.30	1.39	0.26	0.44	0.10
AA2024-T3	89.16	0.17	2.92	7.00	0.75	-	-	-	-	-

**Table 2:** Parameters of post weld heat treatment process.

Parameter	Symbol	Unit	Value		
			−1	0	1
Solid solution time	A	hr	0.5	2.25	4
Solid solution temperature	B	°C	460	480	500
Aging time	C	hr	6	15	24
Aging temperature	D	°C	120	150	190

**Table 3:** Design matrix and experimental results of PWHT process.

StdOrder	RunOrder	CenterPt	Blocks	Factor				Response	
				A (hr)	B (°C)	C (hr)	D (°C)	UTS (MPa)	EL (%)
26	1	1	1	4	500	24	120	310.00	6.20
20	2	1	1	4	460	6	120	245.23	6.02
17	3	0	1	2.25	480	15	155	258.96	5.52
29	4	1	1	0.5	500	6	190	327.91	7.16
11	5	1	1	0.5	500	6	190	302.04	7.40
32	6	1	1	4	460	24	190	229.92	5.23
25	7	1	1	0.5	500	24	120	233.72	4.65
2	8	1	1	4	460	6	120	261.00	6.19
22	9	1	1	4	500	6	120	306.10	6.10
12	10	1	1	4	500	6	190	248.63	4.60
4	11	1	1	4	500	6	120	314.75	6.12
21	12	1	1	0.5	500	6	120	310.25	5.81
7	13	1	1	0.5	500	24	120	239.85	4.99
34	14	1	1	4	500	24	190	240.36	4.78
14	15	1	1	4	460	24	190	248.17	5.42
18	16	0	1	2.25	480	15	155	283.33	5.39
35	17	0	1	2.25	480	15	155	265.48	5.28
33	18	1	1	0.5	500	24	190	285.17	5.91
23	19	1	1	0.5	460	24	120	289.33	6.90
31	20	1	1	0.5	460	24	190	241.26	5.09
13	21	1	1	0.5	460	24	190	215.07	5.03
6	22	1	1	4	460	24	120	277.50	5.92
5	23	1	1	0.5	460	24	120	281.33	6.50
19	24	1	1	0.5	460	6	120	239.90	6.58
8	25	1	1	4	500	24	120	318.38	6.16
15	26	1	1	0.5	500	24	190	282.13	5.49
16	27	1	1	4	500	24	190	231.02	4.35
28	28	1	1	4	460	6	190	285.21	7.15
30	29	1	1	4	500	6	190	251.00	4.40
9	30	1	1	0.5	460	6	190	269.97	7.20
3	31	1	1	0.5	500	6	120	333.10	6.20
26	1	1	1	4	500	24	120	310.00	6.20
20	2	1	1	4	460	6	120	245.23	6.02
17	3	0	1	2.25	480	15	155	258.96	5.52
29	4	1	1	0.5	500	6	190	327.91	7.16
11	5	1	1	0.5	500	6	190	302.04	7.40

A—solid solution time; B—solid solution temperature; C—aging time; D—aging temperature.

**Table 4:** ANOVA table for the UTS response.

Source	DF	Adj SS	Adj MS	F-Value	*p*-Value
Model	15	31,051.0	2070.07	22.93	0.000
Linear	4	9372.2	2343.05	25.96	0.000
A	1	63.3	63.31	0.70	0.412
B	1	4485.7	4485.70	49.69	0.000
C	1	2874.9	2874.90	31.85	0.000
D	1	1948.3	1948.28	21.58	0.000
2-Way Interactions	6	8416.5	1402.74	15.54	0.000
3-Way Interactions	4	12,114.8	3028.70	33.55	0.000
4-Way Interactions	1	1147.6	1147.56	12.71	0.002

DF = degree of freedom; Adj SS = Adjusted sums of squares; Adj MS = Adjusted mean squares; F-value = mean sum-of-squares for regression/mean sum-of-squares for residual; *p*-value = the smallest level of significance at which the data are significant.

**Table 5:** ANOVA table for the EL response.

Source	DF	Adj SS	Adj MS	F-Value	*p*-Value
Model	15	25.5379	1.70253	16.58	0.000
Linear	4	10.5355	2.63388	25.64	0.000
A	1	1.4285	1.42847	13.91	0.001
B	1	3.1922	3.19223	31.08	0.000
C	1	5.6456	5.64564	54.96	0.000
D	1	0.2692	0.26919	2.62	0.121
2-Way Interactions	6	6.3582	1.05969	10.32	0.000
3-Way Interactions	4	8.6372	2.15929	21.02	0.000
4-Way Interactions	1	0.0071	0.00705	0.07	0.796

**Table 6:** The final hyperparameter set of KNN for EL and UTS models.

Random State	Weights		n Neighbors		Leaf Size		*p*	
	UTS Model	EL Model	UTS Model	EL Model	UTS Model	EL Model	UTS Model	EL Model
1	distance	distance	4	2	3	5	3	1
3	uniform	distance	1	2	1	3	1	2
5	uniform	distance	1	4	9	5	2	3
7	distance	distance	2	2	10	9	1	1
9	distance	uniform	3	1	2	1	2	3

**Table 7:** The final hyperparameter set of SVR for EL and UTS models.

Random State	C		Epsilon		Kernel	
	UTS Model	EL Model	UTS Model	EL Model	UTS Model	EL Model
1	82	1	0.01	0.01	rbf	rbf
3	2	155	0.07	0.06	rbf	rbf
5	178	122	0.01	0.08	rbf	rbf
7	103	139	0.05	0.09	rbf	rbf
9	2	129	0.02	0.06	rbf	rbf

**Table 8:** The final hyperparameter set of DT for EL and UTS models.

Random State	Max Depth		Min Samples Split		Min Samples Leaf	
	UTS Model	EL Model	UTS Model	EL Model	UTS Model	EL Model
1	6	5	0.1	0.1	1	1
3	7	50	0.8	0.6	1	3
5	4	2	0.8	0.8	1	3
7	8	5	0.2	0.2	1	1
9	10	1	0.1	0.4	1	1

**Table 9:** The final hyperparameter set of RF for EL and UTS models.

Random State	n Estimators		Max Depth		Min Samples Split	
	UTS Model	EL Model	UTS Model	EL Model	UTS Model	EL Model
1	46	5	4	9	0.1	0.1
3	1	3	6	6	0.4	0.1
5	3	8	8	5	0.1	0.1
7	13	6	9	3	0.1	0.1
9	2	3	8	4	0.4	0.6

**Table 10:** Average MSE and MAE for different ML techniques.

Model	Average MSE				Average MAE			
	SVR	DT	KNN	RF	SVR	DT	KNN	RF
UTS	0.03748	0.06455	0.03000	0.05591	0.15961	0.21877	0.1347	0.19255
EL	0.02072	0.03971	0.01659	0.04228	0.10422	0.14911	0.09563	0.16347

**Table 11:** The optimum PWHT condition results from a single objective (random state = 5).

Objective	Algorithm	Optimal Parameter				Predicted Value
		A (hr)	B (°C)	C (hr)	D (°C)	
UTS (MPa)	SVR-DE	0.50	500.00	6.00	133.87	325.16
	SVR-PSO	0.50	500.00	6.00	133.87	325.16
	SVR-GA	0.50	499.91	6.02	133.78	325.10
EL (%)	SVR-DE	0.50	476.44	6.00	190.00	7.30
	SVR-PSO	0.50	476.43	6.00	190.00	7.30
	SVR-GA	0.51	475.85	6.00	189.81	7.30

**Table 12:** Hypervolume measure of all combinations.

Combination	Hypervolume
SVR-DE	12.004
SVR-PSO	12.043
SVR-GA	12.607

## Data Availability

Data used in this study are available upon reasonable request to the corresponding author.
